# Finding the right size for a group

**DOI:** 10.7554/eLife.63871

**Published:** 2020-11-10

**Authors:** Marlee Tucker

**Affiliations:** 1 Department of Environmental Science, Radboud UniversityNijmegenNetherlands; 2 Institute for Wetland and Water Research, Radboud UniversityNijmegenNetherlands

**Keywords:** collective behaviour, collective movement, group living, movement ecology, social behaviour, group size, Other

## Abstract

Vulturine guineafowl range over larger areas, explore more new places and are more likely to reproduce when they live in groups of intermediate size.

**Related research article** Papageorgiou D, Farine D. 2020. Group size and composition influencecollective movement in a highly social terrestrial bird. *eLife* **9**:e59902. doi: 10.7554/eLife.59902

Think about the last time you were out in a group of people who were deciding where to eat: some members of the group likely had strong opinions about where to go, with others being happy to go with the flow. However, balancing everyone’s opinions without spending hours in discussions or losing people in the process can be tricky, especially in large groups.

In the wild, animals also face similar challenges. Living together provides benefits such as sharing information on where to find food, and providing better protection from predators as there are more individuals to keep watch over the group (Krause and Ruxton, 2002; Kao and Couzin, 2014; Majolo and Huang, 2017). There are also negatives associated with being in a group: inidividual members may compete with each other for resources, and as the group gets bigger it can become harder to maintain coordination (Kutsukake and Clutton-Brock, 2008; Strandburg-Peshkin et al., 2015). However, it is not clear if there is an optimal size that balances the costs and benefits of living together.

Now, in eLife, Danai Papageorgiou and Damien Farine – who are based at the Max Planck Institute of Animal Behaviour, the University of Konstanz, the Kenya Wildlife Service and the National Museums of Kenya – report how group size influenced the movements of wild birds called vulturine guineafowl (Papageorgiou and Farine, 2020). These birds – which are terrestrial in nature – are widely used for studying collective behaviour because they are highly social, form stable groups and often interact with other groups (e.g., for mating and sharing information). Papageorgiou and Farine fitted GPS tracking devices to a total of 58 birds from 21 different groups, and collected data on the size of each bird's 'home range' (that is, the area it covers to find food, to care for its young and to mate), the distance travelled per day, and how often groups re-visited an area. They also counted the number of chicks in each group to obtain an estimate of the group’s fitness.

Papageorgiou and Farine found that intermediate-sized groups – which contained between 33 and 37 birds – had larger home ranges and tended to explore more new places than smaller and larger groups (Figure 1). This is due to the balance between the benefit of increasing group size for navigation (more information about the landscape) and the costs of movement coordination (keeping everyone together) in large groups. The results also showed that groups of intermediate size had more chicks, meaning they have a higher level of fitness than smaller or larger groups. This higher fitness suggests that intermediate-sized groups may be the most effective at using the areas and resources available to them, indicating there is an optimal group size for collective movement.

**Figure 1.**
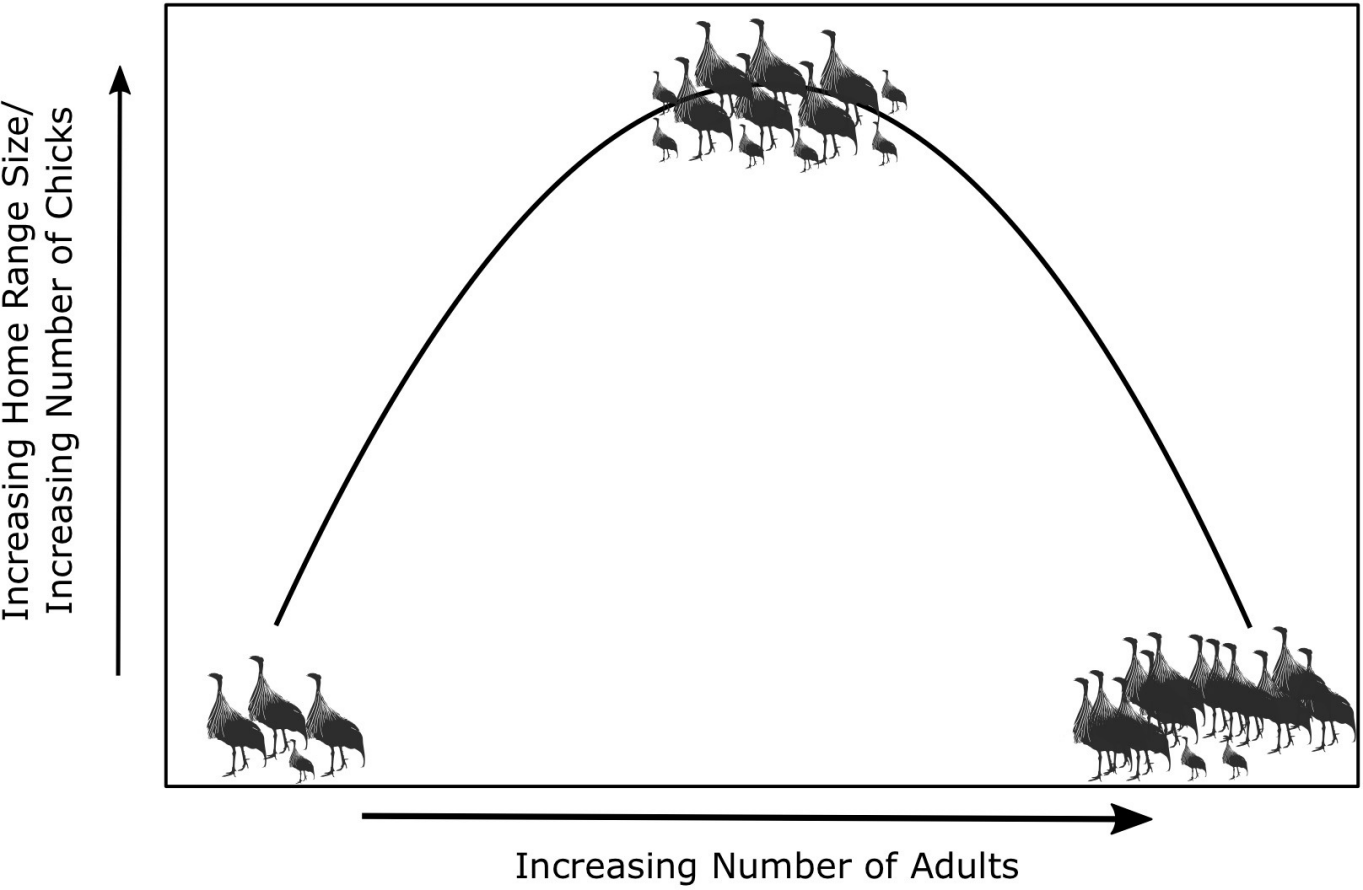
What is the optimal size for a group of vulturine guineafowl? Vulturine guineafowl are social birds that live in groups. Papageorgiou and Farine studied how the size of the home range of these birds, and the number of chicks in each group, varied with the number of adults in a group. They found, as illustrated schematically here, that groups containing an intermediate number of adults (between 33 and 37 birds in this case) have larger home ranges and more chicks than smaller and larger groups, which suggests that intermediate-sized groups benefit from increased fitness.

Papageorgiou and Farine found that most of the groups they studied were smaller or larger than this optimum size. While fitness is maximised in groups of intermediate size, it is difficult to maintain because the number of individuals may fluctuate due to reproduction and immigration (Grueter et al., 2020). Notably, when the intermediate-sized groups had chicks, their home range size decreased. This is because the chicks are more vulnerable to predators, so groups tend to keep under cover and limit their movements. There is a potential trade-off here between those individuals who have successfully reproduced and want to maximise the survival chances of their chicks, and those who did not reproduce and may benefit from having a larger home range size and access to a wider range of resources (Papageorgiou et al., 2019).

The latest work could be taken forward in a number of ways. First, these data were collected during specific seasons with similar weather conditions so that the data for different groups could be compared. This raises the question of whether the benefits associated with intermediate group size are consistent across all seasons, even when resources such as food and water are limited. Second, it would be interesting to explore if similar effects are found across different taxa and landscapes, such as the tropics versus temperate regions, where seasons and resources differ. Finally, guineafowl groups are not territorial animals, and it would be interesting to study what happens when groups of animals are more defensive of their habitats. If competition between groups increases, the areas available would be reduced and groups may spend more energy on defending their territory, in which case it may be better to have a larger sized group (Mosser and Packer, 2009).

These findings shed new light on how the size and composition of groups can shape the movement patterns of animals. This type of integrated approach, using long-term tracking data, is essential to gain a better understanding of the mechanisms of collective behaviour and will be useful for the conservation of vulturine guineafowl and other social species.
